# First record of *Obeza nigromaculata* (Cameron, 1884) (Hymenoptera, Eucharitidae, Eucharitini) from Colombia, with one nomenclatural act for the species

**DOI:** 10.3897/BDJ.14.e176471

**Published:** 2026-04-21

**Authors:** Mónica Lizeth Rivas-Quezada, Jean Gamboa, Francisco Serna

**Affiliations:** 1 Universidad de la Amazonia, Laboratorio de Entomología Universidad de la Amazonia - LEUA, Grupo de Investigación en Entomología Universidad de la Amazonia (GIEUA), Florencia, Colombia Universidad de la Amazonia, Laboratorio de Entomología Universidad de la Amazonia - LEUA, Grupo de Investigación en Entomología Universidad de la Amazonia (GIEUA) Florencia Colombia; 2 Universidad Nacional de Colombia, Sede Bogotá, Museo Entomológico UNAB, Grupo de Investigación en Sistemática de Insectos Agronomía - SIA, Bogotá, Colombia Universidad Nacional de Colombia, Sede Bogotá, Museo Entomológico UNAB, Grupo de Investigación en Sistemática de Insectos Agronomía - SIA Bogotá Colombia

**Keywords:** agroecosystem, cacao, lectotype, wasp

## Abstract

**Background:**

*Obeza nigromaculata* (Cameron, 1884) is recorded for the first time from South America and Colombia, in a cacao-producing agroecosystem of the south-eastern region of the country. In addition, a type specimen from the Natural History Museum, London, is designated as the lectotype of the species.

**New information:**

We provide detailed morphological characters and photographic documentation for the species *Obeza nigromaculata* (Cameron, 1884). One specimen of the species is recorded in an agroforestry system of *Theobroma cacao* L. (Malvaceae) - Cacao, *Juglans neotropica* (Juglandaceae) – Black cedar and *Musa* x *paradisiaca* (Musaceae) - Plantain.

## Introduction

Eucharitidae include 65 genera and 531 species grouped in the subfamilies Akalapinae (1 genus, 2 species), Gollumiellinae (2 genera, 12 species), Oraseminae (13 genera, 140 species) and Eucharitinae (49 genera, 377 species) (UCD Community 2023). Within Eucharitinae, Eucharitini is recognised as a monophyletic group that includes 46 genera and 350 species with worldwide distribution (UCD Community 2023, Heraty et al. 2025).

Eucharitidae is the only family of insects known to parasitise ants exclusively (Murray et al. 2013). Ants have been considered a natural group of almost 100 million years old and Eucharitidae arose approximately 50 million years after the major ant lineages were already established and diversifying (Moreau 2011, Murray et al. 2013, Fernández et al. 2021). In the Neotropical Region, genera in the tribe Eucharitini include *Dicoelothorax* Ashmead, 1899, *Dilocantha* Shipp, 1894, *Isomerala* Shipp, 1894, *Lophyrocera* Cameron, 1884, *Obeza* Heraty, 1985 and *Pseudochalcura* Ashmead, 1904, which are parasitoids of the immature stages of various ant species (Hymenoptera, Formicidae) of the subfamilies Ectatomminae, Formicinae and Ponerinae (Heraty 2002, Lachaud and Pérez-Lachaud 2012).

Based on a morphological phylogenetic analysis, *Obeza* Heraty, 1985 is considered a sister group of *Lophyrocera* Cameron, 1884 within the *Stilbula* clade, based on the presence of short bifurcating frenal processes, broad lateral extensions from the propodeum and unpronounced callus (Heraty 2002). Species of *Obeza* are distributed in the Neotropical and Nearctic Regions (Heraty 1985, Heraty 2002). Individuals can be separated from other genera of Eucharitini by "flagellomeres simple; propodeal processes blunt; callus and process with strong laminate carinae, rarely rugose; body yellow or orange with brown to black patterns, head black or dark cyaneous, rarely almost entirely black; hind tibia with two spurs" (Heraty 2002). The genus includes the following species: *O. floridana* (Ashmead, 1888), *O. grenadensis* (Howard, 1897), *O. maculata* (Westwood, 1874), *O. meridionalis* (Kirby, 1889), *O. nigriceps* (Ashmead, 1904), *O. nigromaculata* (Cameron, 1884), *O. semifumipennis* (Girault, 1911) and *O. septentrionalis* (Brues, 1907) (Heraty 1985, Santis and Fidalgo 1994).

Most interactions between *Obeza* species, their plant hosts and their ant hosts remain unknown. To date, only *O. floridana* has been recorded as a parasitoid of *Camponotus floridanus* (Buckley, 1866) (Davis and Jouvenaz 1990), although the pupa of a species from Dominica (possibly *O. grenadensis*) was found in the pupal cocoon of *Camponotus* (Heraty, unpublished). Regarding the interaction with host plants, *O. floridana* were found to oviposit into fruits of *Vaccinium corymbosum* L. (Ericaceae) and an undescribed species from Mexico oviposited into similar fruits of a species of Boraginaceae (Heraty and Barber 1990).

Knowledge of the distribution of parasitoid species contributes to our understanding of the natural biological control they exert on other insect populations present in the ecosystems and agroecosystems. *Obeza nigromaculata* has only been recorded from the type locality in Nicaragua (Cameron 1884, Schmiedeknecht 1909, Peck 1951, Heraty 1985, Heraty 2002). Accordingly, the aim of this work is to document the first record of *Obeza nigromaculata* (Hymenoptera, Eucharitidae) from Colombia.

## Materials and methods

In the Department of Huila in Colombia, 127 cacao plantations were sampled in a search for parasitoid wasps using an entomological sweep net. Specimens collected were placed in 30 ml plastic bottles containing ethyl alcohol [96%] and transported to Laboratorio de Entomología Universidad de la Amazonia (LEUA) in Florencia (Caquetá, Colombia). Curatorship of specimens, which were point-mounted, was carried out following the protocols established in the LEUA insect collection.

Amongst the collected samples, a specimen was found to correspond to the genus *Obeza*. The taxonomic identification to the genus level was carried out based on Heraty (1985). The only individual (male) that was collected corresponds to *O. nigromaculata*, which was identified by comparing the specimen to the original description of the species published by Cameron (1884) and photographs of the lectotype deposited at the Natural History Museum, London (BMNH).

The dry aedeagus was extracted employing forceps and pins. After dissecting and imaging the aedeagus, it was placed into a microvial attached to the bottom of the specimen pin. General morphological terminology follows Heraty et al. (2018). The specimen was housed in the LEUA Collection.

The preserved *O. nigromaculata* specimen was studied with an Olympus SZ61 stereomicroscope at 90x magnification. Images and measurements were taken using a LEICA M205A stereomicroscope and a HITACHI TM4000Plus II environmental scanning electron microscope. Antenna, labrum, prosternum and propleura and the genitalia were drawn employing the Infinite Painter v. 7.1.18 software. A distribution map of the species was designed using the software QGIS v. 3.26.2. All figures were prepared by utilising the software Photoshop 2023 v. 24.0.

The abbreviations used in this work are: aed = aedeagus, cly = clypeus, dig = digitus, F1 = flagellomere 1, F2 = flagellomere 2, lmd = left mandible, mdl = marginal digit of labrum, msl = marginal seta of labrum, par = paramere, ped = pedicel, phb = phallobase, pl_1_ = propleuron, ps = subapical parameral sensillum, rad = radicle, rmd = right mandible, s_1_ = prosternum, scp = scape, tor = torulus.

**Figure 1.**
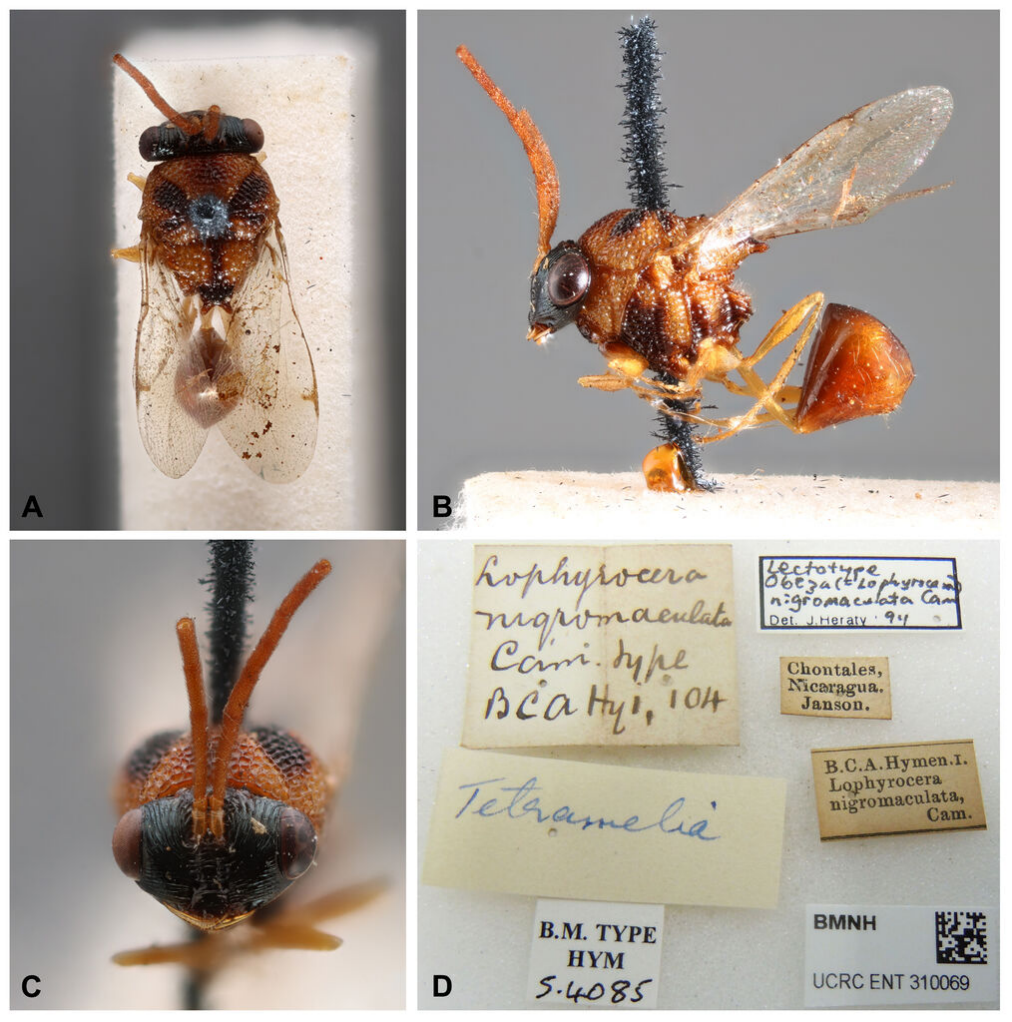
*Obeza nigromaculata* (Cameron, 1884) (Eucharitini), lectotype, male, deposited in BMNH, catalogue number NHMUK 013457788, photographs provided by Natalie Dale-Skey, 2025 of ©The Trustees of the Natural History Museum, London and made available under the Creative Commons License 4.0 https://creativecommons.org/licenses/by/4.0/. **A** Habitus, dorsal view; **B** Habitus, lateral view; **C** Head, anterodorsal view; **D** Labels.

*Obeza nigromaculata* was described by Peter Cameron in 1884 based on a male, but without designation of a holotype and no indication of how many specimens the description was based on. In 1994, John M. Heraty compared the original publications by Cameron with a male type specimen available at the BMNH and labelled it as a lectotype, but this was not published. The male is formally designated herein as a lectotype [**Type material.** Lectotype hereby designated: NICARAGUA – Chontales. Janson.; Lophyrocera nigromaculata, Cam. type BCa Hyi, 104 [handwritten]; type B.C.A. Hymen.I. Lophyrocera nigromaculata, Cam.; B.M. TYPE HYM 5.4085; Tetramelia; Lectotype Obeza (= Lophyrocera) nigromaculata Cam. Det. J. Heraty '94; BMNH UCRCENT 310069 (Fig. 1)], in accordance with Article 74.7 of the International Code of Zoological Nomenclature (ICZN 1999) and Declaration 44 (ICZN 2003).

## Taxon treatments

### Obeza nigromaculata

(Cameron, 1884)

9D874848-5604-5D24-8467-87EB1A9DCAAD

https://ucd.chalcid.org

*Lophyrocera nigromaculata* Cameron, 1884

#### Materials

- **Type status:** Other material. **Occurrence:** catalogNumber: LEUA-55135; recordedBy: J. Gamboa; individualCount: 1; sex: male; lifeStage: adult; occurrenceID: 1B441011-7C24-559B-95EC-157B1CED5F93; **Taxon:** taxonID: urn:lsid:biosci.ohio-state.edu:osuc_names:275502; scientificName: Obeza nigromaculata; **Location:** country: Colombia; stateProvince: Huila; locality: Vda. Los Cauchos, Fca. La Cabaña; verbatimElevation: 865 m; locationRemarks: label transliteration: "Colombia, Huila, Neiva, 2021.10.25, L. Andrade"; [865 m, 02°52'26"N 75°08'39"W, 2021.10.25, collected employing an entomological net in an agroforestry system of Theobroma cacao L. (Malvaceae) - Cacao, Juglans neotropica (Juglandaceae) – Black cedar, and Musa paradisiaca (Musaceae) - Plantain]; verbatimCoordinates: 02°52'N 75°08'E; decimalLatitude: 3; decimalLongitude: 75; georeferenceProtocol: GPS; **Identification:** identifiedBy: J. Gamboa; dateIdentified: 2025; **Event:** eventID: urn:lsid:biosci.ohio-state.edu:osuc_occurrences:LEUA__55135; samplingProtocol: entomological net; eventDate: 25/10/2021; **Record Level:** language: Spanish; collectionID: urn:lsid:biocol.org:col:55135; collectionCode: Insects; basisOfRecord: PreservedSpecimen

#### Description

**Figure 2.**
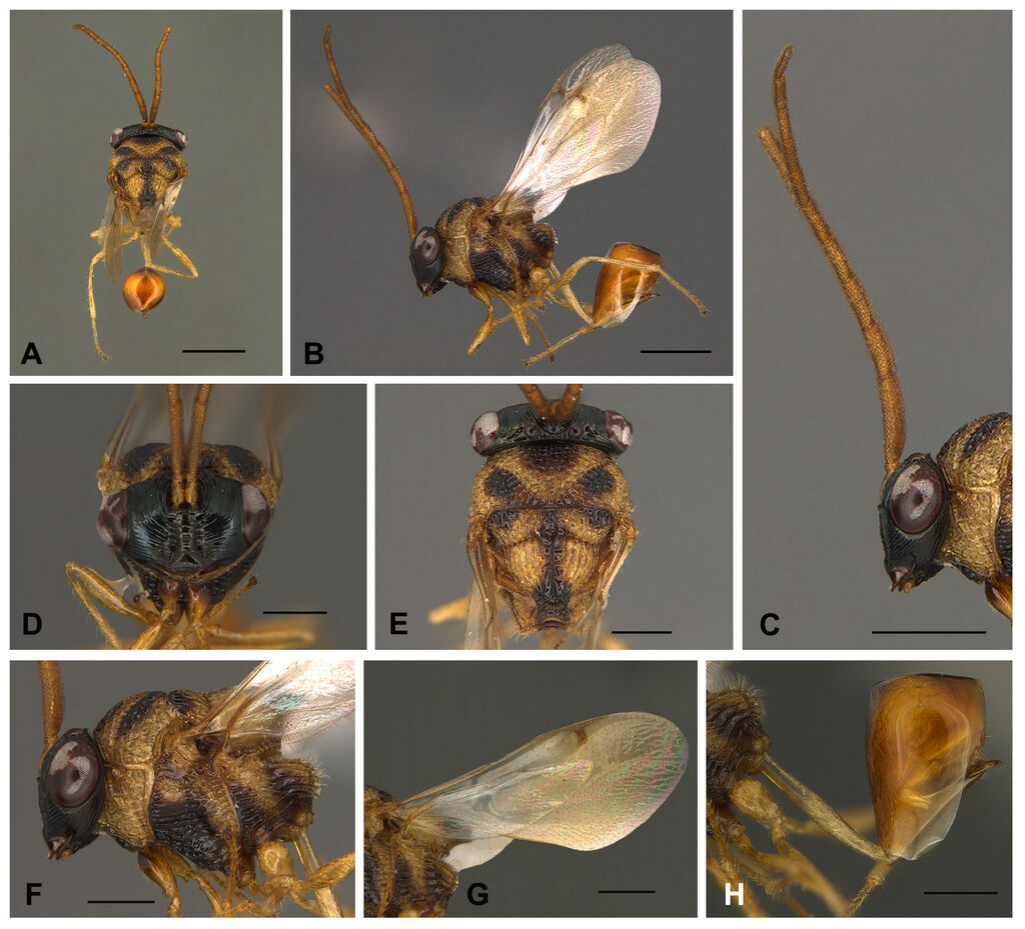
*Obeza nigromaculata* (Cameron, 1884) (Eucharitini) deposited at LEUA, male. **A–B** Habitus: **A** Dorsal view; **B** Lateral view. **C–D** Head: **C** Lateral view; **D** Anterior view. **E–F** Mesosoma: **E** Dorsal view; **F** Lateral view; **G** Wings; **H** Petiole and gaster, lateral view. Scale bars: 1 mm (**A–C**); 0.5 mm (**D–H**).

**Figure 3.**
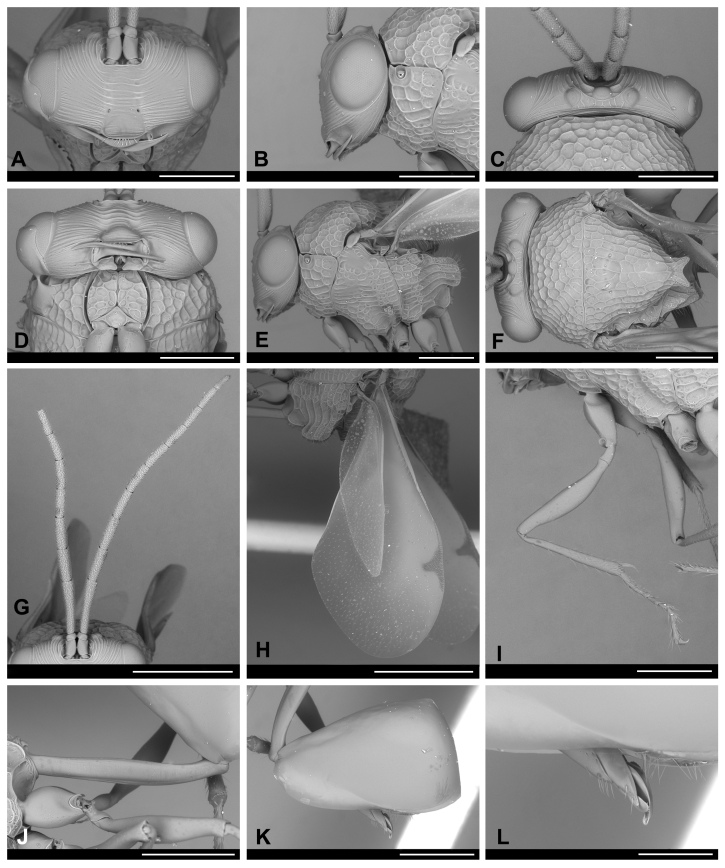
*Obeza nigromaculata* (Cameron, 1884) (Eucharitini) deposited at LEUA, male. **A–C** Head: **A** Anterior view; **B** Lateral view; **C** Dorsal view. **D** Prosternum and propleura; **E–F** Mesosoma: **E** Lateral view; **F** Dorsal view. **G** Antenna; **H** Wings; **I** Fore leg; **J–L** Metasoma, lateral view: **J** Petiole; **K** Gaster; **L** Genitalia. Scale bars: 200 μm (**L**); 500 μm (**A–F, I–K**); 1.0 mm (**G–H**).

**Figure 4.**
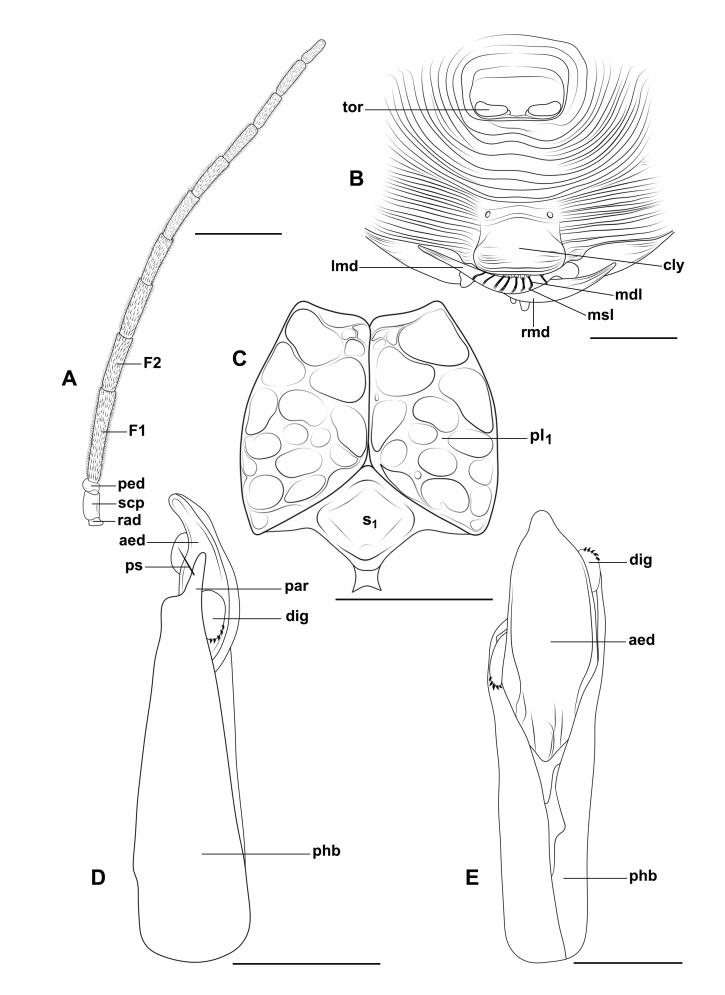
*Obeza nigromaculata* (Cameron, 1884) (Eucharitini) deposited at LEUA, male. **A** Antenna; **B** Frons, clypeus and labrum; **C** Prosternum and propleura; **D–E** Genitalia: **D** Lateral view; **E** Dorsal view. Scale bars: 100 μm (**E**); 150 μm (**D**); 250 μm (**B–C**); 0.5 mm (**A**).

**Identification.** Features agreeing with the original description of *O. nigromaculata*: Body length 4.8 mm. Body yellowish-brown; head black; mesosoma with black spots; petiole and legs yellowish-brown; wings hyaline. Two longish, large, disc-shaped patches over the posterior legs, a patch in front of the mesonotum, two large patches behind this one that come together in the mesial line, a roundish small patch outside of these patches and, touching them at their apex, a somewhat triangular patch (which is prolonged as a broad line to the end of the scutellum, where it becomes broad), all black; metanotum lower side black. Gaster brown, slightly darker posteriorly. Face with striae radiating laterally and dorsally from clypeal region. Occiput with strong dorsal carina. Supraclypeal area bordered by deep foveate subantennal sucli; clypeus smooth medially. Antennae long and cylindrical; scape short, flagellomere 1 1.7x as long as F2 length. Mesosoma with strongly rugose-areolate. Mesoscutellum apex with two blunt teeth, each 2.0x as long as wide. Metapleuron with five or six large longitudinal striations, lateral aspect with two large blunt processes. Petiole long and slender, longer than the hind femora. The Colombian specimen and the lectotype deposited at the BMNH are similar in shape, colouration and sculpture (Figs 1, 2, 3, 4). However, the former has a black mark on pronotum anterior aspect that is clearly delimited and darker; and a gaster darker brown-orange, clearer than the mesosoma (Fig. 2).

The Colombian specimen that is identical to the type has head glabrous; labrum with eight marginal digits with spaculate terminal setae of length subequal to the maximum width of the mandible; antenna with 12 segments, scape and pedicel glabrous, flagellum with short and abundant setae; mesosoma mostly glabrous with some short and slender setae dorsally, with very few on the pronotum ventral edge, prosternum rhomboid (ventral view) and glabrous, propleura conical and glabrous, metapisternum with long setae dorsomedially and dorsoposteriorly; fore wing: submarginal vein with few suberect setae, stigmal vein with dense setae and wing disc with microtrichia, denser in distal portion; hind wing: hamuli with four hooks, posterior margin with erect and suberect setae, denser microtrichia in distal half; legs with coxa, trochanter and femur glabrous, tibia with setae of different sizes, suberect and subdecumbent denser towards the apex, some as long as tibia width in the middle, apex with setae longer, thicker and stronger, tarsus with setae longer than the diameter of tarsomeres; petiole and gaster mostly glabrous, most posterior metasomal sternite with several erect setae (Fig. 3, Fig. 4). Phallobase subcylindrical, slightly wider in distal portion, 4.1x as long as width; aedeagus oval with cone-shaped apex; digitus laminar and subrounded with five digital spines (Fig. 3, Fig. 4).

#### Distribution

**Figure 5.**
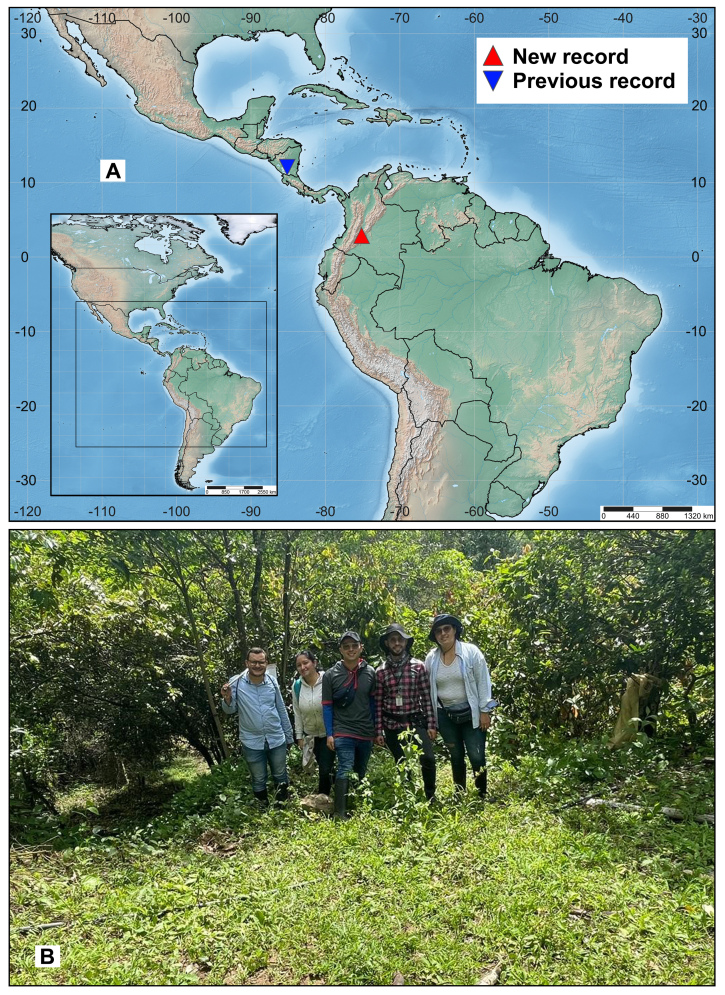
Ecological aspects of *Obeza nigromaculata* (Cameron, 1884) (Eucharitini). **A** Geographical distribution; **B** Habitat.

**Previously known distribution.** Nicaragua (Chontales) (Fig. 5) (Cameron 1884, Schmiedeknecht 1909, Peck 1951, Heraty 1985, Heraty 2002). A record from Arizona reported by Ashmead (1892) was considered erroneous by Heraty (2002).

#### Taxon discussion

To date, there are only two records of *O. nigromaculata*, so knowledge of the biogeography distribution of the species is restricted. Despite the ecological importance of the species as a biological controller of ants, knowledge of its biology (immature stages and female) and ecology is very limited.

Cacao-producing agroforestry systems in Colombia are increasing rapidly. New insights into these environments by employing new sampling efforts, which focus on fruit collection (mainly barries) and a variety of surrounding vegetables would allow us to find and determine the host ant of *O. nigromaculata*. Previous research indicates that berry sampling would facilitate the possible tracking and identification of the host ant species (Heraty and Barber 1990, Heraty et al. 2025).

## Discussion

Most *Obeza* species were described in the genera *Lophyrocera* Cameron, 1884, *Stilbula* Spinola, 1811, *Schizaspidia* Westwood, 1835, *Orasema* Cameron, 1884 and *Tetramelia* Kirby, 1889. *Lophyrocera* is the most morphologically similar to *Obeza*, but distinguished from *Obeza* by having truncate or pectinate versus simple antenna, truncate versus cylindrical petiole and an abrupt shelf-like versus angulate gena (Heraty 2002). Currently, there is a precise morphological delimitation for *Obeza*, but there are no taxonomic keys for the identification of the species in this genus (Heraty 1985, Heraty 2002).

The lectotype was collected in the locality of Chontales in Nicaragua (Central America) (Fig. 5). This work presents the first record of *O. nigromaculata* for South America and Colombia (Fig. 5). As there are only two recorded locations for *O. nigromaculata* in the Neotropical Region, information is considered very limited to understand the biogeographical aspects of the species and genus.

The Neotropical Region includes 137 genera and approximately 3,100 species of ants (Fernández et al. 2021). In agroecosystems, ants can significantly impact agricultural production and yield through interactions with other organisms (Sankovitz et al. 2024). Species of Eucharitini are parasitoids of the immature stages of ants of the genera *Camponotus* Mayr, 1861, *Dinoponera* Roger, 1861, *Ectatomma* Smith, 1858, *Gnamptogenys* Roger, 1863, *Hypoponera* Santschi, 1938, *Lasius* Fabricius, 1804, *Odontomachus* Latreille, 1804, *Pachycondyla* Smith, 1858, *Polyrhachis* Smith, 1857 and *Typhlomyrmex* Mayr, 1862 (Lachaud and Pérez-Lachaud 2012).

At least eight species of *Camponotus* have been recorded as hosts for species in the genera *Lophyrocera*, *Obeza*, *Pseudochalcura* and *Stilbula* (Wheeler 1907, Fahringer and Tölg 1912, Clausen 1923, Clausen 1941, Heraty 1986, Torréns et al. 2008, Davis and Jouvenaz 1990, Heraty and Barber 1990, Heraty 2002, Heraty et al. 2009).

Only one specimen of *O. nigromaculata* was found in the agroforestry system dominated by *T. cacao* (Fig. 5). These agroecosystems feature a high richness and abundance of ants, associated with ecological functions, such as phytophagy on cacao trees, pest predation or as vectors of plant pathogens (Delabie 1990, Bisseleua et al. 2017). Future studies should focus on identifying *Obeza* species that interact with ant populations in these agroecosystems.

## Supplementary Material

XML Treatment for Obeza nigromaculata
